# Epidemiology of Prothrombin G20210A Mutation in the Mediterranean Region

**DOI:** 10.4084/MJHID.2011.054

**Published:** 2011-11-28

**Authors:** Mehrez M. Jadaon

**Affiliations:** Department of Medical Laboratory Sciences, Faculty of Allied Health Sciences, Kuwait University, Kuwait

## Abstract

There are many genetic and acquired risk factors that are known to cause venous thromboembolic disorders (VTE). One of these is the Prothrombin G20210A mutation, which has been identified in 1996. Prothrombin G20210A mutation causes higher levels of the clotting factor prothrombin in the blood of carriers, which creates a higher tendency towards blood clotting (hypercoagulability), and therefore the carriers become at higher risk of developing VTE. High prevalence of Prothrombin G20210A mutation was reported in Caucasian populations, but the prevalence was almost absent in non-Caucasians. That was most obvious in countries of South Europe and the Mediterranean region. This review article discusses Prothrombin G20210A mutation, how it causes VTE, the origin of the mutation, and its distribution worldwide with special concentration on the Mediterranean area.

## Introduction

Venous thromboembolic disorders (VTE) are serious disorders accounting for high morbidity and mortality rates with an annual incidence of 1/1000.^1^–^4^ Many genetic and acquired risk factors were identified to cause VTE including Factor V Leiden mutation, genetic deficiencies of proteins C, S and antithrombin, lupus anticoagulants, pregnancy, use of contraceptives, major surgeries, cancer, inflammations, and Prothrombin G20210A mutation. This review article focuses on Prothrombin G20210A mutation, its pathophysiology, prevalence and origin, with a special concentration on this mutation in the Mediterranean region.

## Role of Prothrombin in the Coagulation System

In case of blood vessel injury, blood coagulation is initiated through a cascade of chemical reactions to form a blood clot to block the injured blood vessel and prevent blood loss. Several enzymes and proteins, generally known as blood clotting factors, are involved in blood coagulation, a very important one is thrombin (clotting factor II). Thrombin is usually produced in the liver in an inactive form called prothrombin, which circulates in the blood until being activated in case of injury. Potrhombin gets activated into thrombin by another clotting factor called activated factor X. The main function of thrombin is to convert fibrinogen (clotting factor I) into a fibrin clot that blocks the injured blood vessel. In fact, thrombin is a very robust enzyme that plays a major role in the coagulation system by activating many clotting factors and other elements of the coagulation system like the blood platelets. Thrombin is usually under careful monitoring by an inhibitor called antithrombin (AT), which down-regulates thrombin after clot formation and prevents accidental formation of thrombin in sites away from injured vessels.^5^,^6^ This is very crucial to prevent the formation of unnecessary clots inside intact blood vessels. The formation of such intravascular clots predisposes to the development of VTE.

## Prothrombin G20210A Mutation

Because of the importance of thrombin in the coagulation system, genetic or acquired deficiency of prothrombin usually causes impaired clotting and therefore bleeding problems (hemophilia). On the contrary, if prothrombin is produced in higher quantities in the blood, this is expected to cause an increased tendency towards blood clotting, a condition known as “hypercoagulability”, which usually manifests clinically as VTE. It has been demonstrated that prothrombin levels more than 115% of the normal level have 2-fold increased risk of developing VTE.^7^ Poort et al (1996) performed an extensive DNA sequencing on the prothrombin gene (on chromosome 11) for patients with unexplained VTE.^7^ They found a single missense mutation (guanine to adenine; G→A) at nucleotide position 20210, which is present in the 3′ untranslated region of the prothrombin gene. This Prothrombin G20210A mutation is present outside the coding region for prothrombin, and hence it does not affect the actual structure of the prothrombin molecule and it does not affect its function as a strong clotting factor when activated into thrombin. However, Prothrombin G20210A mutation was found to cause elevated levels of blood prothrombin (by one-third above normal; 133%), which is more than the extra 15% needed to develop VTE. Also, it has been proven that Prothrombin G20210A mutation leads to increased mRNA and protein expression for prothrombin.^8^ Moreover, increased prothrombin levels may lead to an increase in a protein called thrombin-activatable fibrinolysis inhibitor (TAFI), which is an inhibitor of the fibrinolysis process. Fibrinolysis is the process by which the blood removes clots. Therefore, an increase in TAFI may disturb the fibrinolysis process and therefore allow for accumulation of clots leading to VTE.^9^,^10^ This is another possible pathophysiological pathway of causing VTE in cases having Prothrombin G20210A mutation with elevated TAFI. All these findings may explain why Prothrombin G20210A mutation may cause hypercoagulability and an increased risk of developing VTE. Such increased risk was reported to be 2 to 4-fold.^7^,^11^–^15^

## Prevalence of Prothrombin G20210A mutation

The prevalence of Prothrombin G20210A mutation in European Caucasians was found to be roughly 3–17% in patients with VTE and 1–8% in healthy controls. That was also true in Caucasians living outside Europe like in the USA, Australia, Brazil and Israel (Table 1). It may be noticed here that the prevalence of Prothrombin G20210A mutation is higher in the Southern European countries than in the Northern countries, in spite of presence of overlapping between the North and South. On the other hand, Prothrombin G20210A mutation was found to be very rare or even absent in Asian and African populations, and in native populations of America (Amerindians) and Australia (Table 1). This was also true when these populations were studied in countries outside their origin like African Americans and Asians living in the USA. The only exception to the above observations is the high prevalence reported in Hispanics and Mexican Mestizos; the latter are descendants of mixed marriages between Europeans and Amerindians. The presence of European genes in such populations may explain the high prevalence of Prothrombin G20210A mutation in these.

High prevalence of Prothrombin G20210A mutation was also reported in populations living close to Europe, namely countries of the Middle East and North Africa. In fact, the prevalence in these countries was very comparable with the prevalence reported in Southern European countries. Therefore, the countries present on the coasts of the Mediterranean Sea, including Southern Europe, may be grouped together sharing the same prevalence of Prothrombin G20210A mutation (Table 2). These countries, 20 in total, have a prevalence of 3–24% in patients with VTE and 1–12% in the general population. No reports could be found in Malta, Syria, Bosnia, Albania and Macedonia. The highest prevalence was found by a study in Egypt in patients with VTE, but the highest among the general populations (healthy controls) was in Palestinians living in Israel (Israeli Arabs). Unfortunately, there were no reports on the prevalence in Palestinian patients with VTE, which kight be higher than the one reported in Egypt, noting that Egypt and Palestine are geographically neighbours.

## Origin of Prothrombin G20210A mutation

The highest prevalence of Prothrombin G20210A mutation in European Caucasians brought up speculations that Prothrombin G20210A mutation might have occurred as a single event in a single Caucasian ancestor and that the current Caucasian carriers of the mutation should have descended from that proposed grandparent. This assumption was supported by a molecular study that found a haplotype to be associated with more than two third of carriers of the mutation compared to one third of non-carriers. This suggests a founder effect, and the mutation was estimated to occur around 24 thousand years ago after the divergence of Africans from Non-Africans and Caucasoids from Mongoloids.^110^ It is tempting to explore this founder haplotype in non-Caucasian carriers of the Prothrombin G20210A mutation in the Middle East and North Africa to see if this founder effect occurred there too. In addition, it may be interesting to study this mutation in the Basque population (in France and Spain), who is thought to be the oldest ethnic group living in Europe. Finding or not finding the mutation in this population may give a hint on the origin of the mutation and to know if it occurred inside or outside Europe. Also, if a future study can prove that Palestinian patients with VTE have the highest prevalence of Prothrombin G20210A mutation (like the general Palestinian population), then this region (Palestine/Israel) may be the place where the mutation has occurred and then spread to Europe and other parts of the Mediterranean region. This region has witnessed a lot of mankind movements since the old ages, since the Neolithic period, and then the Phoenicians who appeared in Lebanon and then cruised to many cities on the Mediterranean coast, followed by the Roman and Greek civilizations, and more recently the Crusaders and Ottomans. Therefore, it is encouraging to try to find certain genetic or chromosomal markers that can help in following the migratory history of manhood in the Mediterranean region which may give a final approach towards determining exactly where the mutation has occurred first and how it spread all over the Mediterranean region.

## Conclusions

The prevalence of Prothrombin G20210A mutation differs in different countries and ethnic groups, being highest in Caucasians, especially those in the Southern Europe, and in the Mediterranean region. Further studies are needed to verify where exactly has the mutation occurred first and how it was carried to other parts of the world.

## Figures and Tables

**Table 1:** Prevalence of Prothrombin G20210A mutation in different populations and countries worldwide.

	Region/Country	VTE patients %	Healthy population %	References
**Caucasians**	North Europe (UK, Ireland, Sweden, Finland, Belarus, Russia, Denmark, Netherlands, Poland, Germany)	6.5	0–2.9	16–26
	South Europe (France, Austria, Spain, Switzerland, Hungary, Italy, Slovenia, Croatia, Serbia, Greece, Turkey, Cyprus)	2.7–17.2	0.7–8.0	16,17, 28–67
	USA	3.2–9.7	1.3–5.0	68–71
	Brazil	---	1.7	72
	Australia	---	4.3	73
	Israel	---	4.0	74
**Africans**	Ivory Coast, Central Africa Republic, Madagascar, Kenya, Mali	---	0	75
	Zaire, Cameron	---	0	72
	USA	1.1–2.2	0–0.3	68,69,70, 71,76,77
	Israel	---	0	74
	Brazil	---	0	72
**Hispanics**	USA	8.9	0.5–2.4	68,70
**Mexican Mestizos**	Mexico	13.5–15.0		78,79
**Asians**	China	0	0	80,81
	Japan		0	72,82,83
	Korea	0	0	68,84,85
	Mongolia		0	75
	Taiwan	0	0	75,86,87
	Southeast Asia (Indonesia, Burma, Cambodia, Thailand, Taiwan, Vietnam, Hong Kong	---	0	75
	India	0	0–0.6	68,75,88–92
	Pakistan, Bangladesh	---	0	88
**Native Americans (Amerindians)**	USA	---	0	68
	Canada	---	0	75
	Brazil	---	0	72,75
	Mexico	---	0	75
**Australian Aboriginals**	Australia	---	0	93
**Australasians**	Papua New Guinea, Vanuatu, Tonga, Micronesia	---	0	75

**Table 2:** Prevalence of Prothrombin G20210A mutation in the Mediterranean countries.

Country	VTE patients %	General healthy population %	References
Lebanon	19.2	1.3–3.6	94–99
Palestine	---	6.5–11.7	74,100
Egypt	23.75	3.33	101
Tunisia	3.2	0–7.4	74,79–99,102–105
Libya	---	2.2	74
Algeria	6.0	1.8	106
Morocco	---	2.4–5.5	74,107–109
Spain	2.7–17.2	2.9–6.5	31–35
France	4.6	1.0–3.1	36–39
Italy	4.3–15.9	2.3–5.7	40–47
Slovenia	5.8–11.3	3.1–4.8	48,49
Croatia	8.0–8.3	2.5–4.0	50–53
Serbia	11.4	2.3–6.0	54–56
Greece	6.8–10.1	2.0–2.7	57–61
Turkey	4.0–10.5	0.7–8.0	62–65
Cyprus	---	2.0–7.8	66,67
